# Preventive plasmapheresis for rituximab related flare in cryoglobulinemic vasculitis

**DOI:** 10.1016/j.jtauto.2023.100194

**Published:** 2023-02-11

**Authors:** Léa Fornero, Tarik Kanouni, Jean-Jacques Tudesq, Camille Pochard, Pauline Verot, Wendy Renier, Ludovic Gabellier, Guillaume Cartron, Philippe Guilpain, Charles Herbaux

**Affiliations:** a CHRU Montpellier: Hématologie Clinique, France; b CHRU Montpellier: Médecine Interne et Maladies Multi-organiques, France; c CNRS UMR 9002 - Institute of Human Genetics, France

**Keywords:** Vasculitis, Croglobulinemia, Plasmpaheresis, Flare, Immunity, Outcome

## Abstract

**Introduction:**

Rituximab monotherapy represents the main therapeutic option for cryoglobulinemic vasculitis (CV) with severe organ involvement. However, initial worsening of the CV, known as rituximab-associated CV flare (=CV flare), has been described and are associated with high mortality rates. The aim of the present study is to evaluate the outcomes of plasmapheresis initiated before or during rituximab treatment, as prevention of CV flare.

**Methods:**

We conducted a retrospecttive study in our tertiary referral center from 2001 to 2020. We have included all patients with CV receiving rituximab and divided them in two groups whether they had flare prevention by plasmapheresis or not. We evaluated rituximab-related CV flare incidence in both groups. CV flare was defined as the onset of a new organ involvement or worsening of the initial manifestations within 4 weeks following rituximab.

**Results:**

Among the 71 patients included, 44 received rituximab without plasmapheresis (control = CT cohort) and 27 received plasmapheresis before or during rituximab treatment (preventive plasmapheresis = PP cohort). PP was given to patients thought to have a high risk of CV flare, with significantly more severe diseases than patients in the CT cohort. Despite this, no CV flare was observed in the PP group. In the other hand, 5 flares occurred in the CT cohort.

**Conclusion:**

Our results show that plasmapheresis is efficient and well tolerated to prevent rituximab-associated CV flare. We believe that our data support the use of plasmapheresis in this indication, especially in patients with high risk of CV flare.

## Highlights

• Cryoglobulinemia vasculitis flare at rituximab initiation is a rare but life threatening side effect that is poorly understood.
• Preventive plasmapheresis is efficient and well tolerated to prevent this phenomenon.
• Patient receiving preventive plasmapheresis before rituximab initiation have an increased time to next treatment.
• Underlying mechanisms differ between type I or II cryoglobulinemias

## 1 Introduction

Cryoglobulins are immunoglobulins (Ig) that precipitate at temperatures below 37 °C. Cryoglobulinemia, that refers to the presence of cryoglobulins in serum, can be asymptomatic or may cause a multi-organic disease known as cryoglobulinemic syndrome or cryoglobulinemia vasculitis (CV) [1]. Cryoglobulins are commonly divided in three categories based on immunoglobulins composition and pathophysiology [2]. Type I cryoglobulins (10–15%) contain isolated monoclonal Ig (mostly IgM but sometimes IgG) and are mainly associated with hematological diseases even in initial stages. Type II and type III cryoglobulins are known as mixed cryoglobulinemia, with Ig having the ability to bind the Fc domain of the IgG, also called rheumatoid factor activity. Most of cryoglobulinemia are asymptomatic and the occurrence of symptoms varies from 2% to 50% depending on series [3]. Clinical manifestations are heterogeneous and due to small-to-medium vessel involvement. The severity of organ involvement is highly variable, from non-functional to life-threatening consequences [4]. The most common manifestations are weakness, renal involvement (proteinuria, renal insufficiency), cutaneous manifestations (purpura, ulcers, livedo, necrosis), neurological symptoms (bilateral and symmetric peripheral neuropathy) and musculoskeletal manifestations (arthralgia or less often arthritis) [5].

CV treatment is reserved to symptomatic disease and always includes the treatment of the underlying disorder [6]: immunochemotherapy for B cell lymphoproliferation (BCL), immunomodulators or proteasome inhibitors for plasma cell disorders (PCD), immunosuppressive therapies for auto-immune disease (AID) or antiviral therapies for post viral diseases (mainly hepatitis C virus (HCV) infection). In severe cases, discontinuing the immune process can be obtain immediately through plasmapheresis or high doses of corticosteroids. In order to reduce the risk of relapse, immunosuppressive treatment (rituximab or cyclophosphamide) may be associated even when no effective treatment of the underlying cause is known [7]. This is how rituximab monotherapy represents one of the main immunosuppressive therapeutic options in CV [[8], [9], [10]] Plasmapheresis, an extra-corporeal plasma exchange designed to remove harmful proteins from patients, is used in active moderate to severe cryoglobulinemia with renal impairment, neuropathy, arthralgia and/or ulcerating purpura [11]. It allows a thorough but transitory elimination of the circulating cryoglobulin complexes in 70–80% of patients. Plasmapheresis could bestow a useful option while awaiting for the background regimen triggering, and it can also be used as maintenance treatment to avoid recurrences [11].

Initial worsening of the CV may occur following rituximab treatment [[12], [13], [14], [15]]. An initial elevation of IgM and/or cryoglobulin levels is observed, sometimes concomitant with autoimmune manifestation clinical worsening and new organ damage. Desbois et al. reported 3.4% of these CV flares in a cohort of 185 patients diagnosed with auto-immune diseases [16] and all of them had type II CV. CV flares occurred after a median time of 8 days upon rituximab infusions. It was associated with severe symptoms in all patients and the subsequent mortality rate was high (57%). Renal involvements and/or underlying BCL seemed to be strong CV flare predicators. More recently, Sy-Go et al. reported 22% of CV flares related to rituximab treatment in a cohort of 64 patients with CV [17] with the same mortality rate (57%). All types of cryoglobulins were concerned and CV flares occurred after a median time of 5.5 days. CV flare management is not well established, and the therapies used in both cohorts were heterogeneous. Described treatments were mostly based on medication such as steroids and chemotherapy, and/or plasmapheresis.

Plasmapheresis for prevention of the IgM flare effect in WM at rituximab initiation has been suggested according to IgM level and/or evidence of hyperviscosity syndrome [6]. By analogy and through clinical observation, it is commonly admitted that CV flare could be thus averted. That is why preventive plasmapheresis is performed on most patients with moderate to severe CV requiring rituximab treatment in Montpellier University Centre. The main goal of the present study is to retrospectively evaluate the incidence rituximab-related flare in CV when preventive plasmapheresis is performed. Secondary objectives are to evaluate treatment responses, intensive care unit (ICU) admissions or renal replacement therapy, overall survival (OS), time to next treatment (TTNT) and plasmapheresis tolerance.

## 2 Patients & methods

### 2.1 Patients

We conducted a retrospective study in our tertiary referral center from 2001 to 2020. We have included all the adult patients with a CV receiving the anti-CD20 chimeric monoclonal antibody rituximab and divided them in two groups depending on whether they received flare prevention by plasmapheresis or not. The inclusion was made upon the patients' digital medical files according to the “Programme de Médicalisation des Systèmes d’Information” coding (PMSI), the French hospital system, based on the Diagnosis Related Groups model, and with the agreement of the ethics committee “Comité Local d’Ethique Recherche” (IRB, n°202000652) and the French personal data protection committee “Commission Nationale de l’Informatique et des Libertés” (CNIL). All patients file were re-analysed by one examiner to assess the correct diagnosis reported on PMSI. We reported all the demographic characteristics and disease statuses before and after treatment.

Among 226 patients extracted from the database, 79 were excluded (64 due to misdiagnosis, 7 because of biologically undemonstrated cryoglobulinemia, and 8 due to lack of information concerning the treatment received). The remaining 147 subjects had cryoglobulinemia vasculitis proven by laboratory evidence but 76 were not treated with rituximab-based therapies. Our work was focused on the 71 remaining patients. The choice between performing PP or not was made on a case-by-case basis, depending on the expected risk of flare effect. In other words, PP was given to patients thought to be at high risk of flare effect. Of note, following a severe flare in 2013 (patient 3), we decided to we decided to be broader in our indications of PP.

### 2.2 Plasmapheresis and rituximab treatments

Plasmapheresis was performed in the apheresis unit of Montpellier University Hospital. Indication, initiation time and frequency of plasmapheresis sessions were initiated by patients' referring physician and discussed with the physician of the apheresis unit. Plasmapheresis duration depended on the objective of plasma volume treated, decided by apheresis physician depending on comorbidities and plasmapheresis tolerance. To obtain the best efficiency and to limit re-circulation effect, it must not exceed 1.4 total plasma volume (TPV) per session. The TPV is calculated with the total blood volume (depending on patient's height and weight) and the hematocrit measured on blood sample. Human albumin (4%) was used for compensation of extracted plasma. It can be mixed with/or replaced by fresh frozen plasma according to fibrinogen level and/or hemorrhage risk. Plasmapheresis machines available in our units are AMICUS™ and COMTEC™ provided by Fresenius SE & Co. KGaA, Bad Homburg vor der Höhe, Germany, and OPTIA SPECTRA™ and SPECTRA™ obtained from Terumo Corporation, Tokyo, Japan. Rituximab indication and regimen (4 weekly 375 mg per m^2^, or 1 g at day 1 and day 15) were decided by the patients' referring physicians and discussed in multidisciplinary meetings. Our center uses either the Mabthera ® (Roche Laboratories, Basel, Switzerland) or Truxima ® (Biogaran, Colombes, France) specialties, according to the current one available in the department at this time.

### 2.3 Definition of flare and plasmapheresis prevention

In the present study, CV flare was defined as the onset of a new organ involvement or as the objective worsening of the initial manifestations within the 4 weeks following rituximab treatment initiation. Plasmapheresis was considered to be a rituximab flare prevention strategy when set up within 4 weeks before or 1 week after the first rituximab infusion, and at least one day before rituximab-related flare first signs. Response to treatment was chosen as the best response regarding organ involvement, according to the referring physician's evaluation. CV biological activity was assessed by measuring C4 plasmatic levels before and after rituximab therapy. Time to next treatment is defined by the time between rituximab first infusion and new treatment initiation. The overall survival is defined by the time between rituximab first infusion and death from all-causes.

### 2.4 Statistical analysis

Primary endpoint was flare incidence after rituximab initiation in the two groups. We used GraphPad Prism 8 software (Dotmatics). Categorical variables are presented with absolute number “n” and percentage “%” in each groupe (n (%)). Fisher's exact test was used to compare them. Continuous variables are presented with median number and interquartile range. To compare frequencies in 2 unpaired groups, we used an unpaired Student *t*-test. Two-tailed nominal P < 0.05 was considered significant. The Kaplan-Meier method was used to estimate OS and TTNT probabilities from initiation of rituximab. Logrank test was used to compare estimates of hazard functions of the two groups at median.

## 3 Results

### 3.1 Main characteristics of control and preventive plasmapheresis cohorts

The PP cohort (preventive plasmapheresis cohort) includes 27 patients, it contains most patients with moderate to severe CV requiring rituximab treatment. The CT cohort (control cohort) includes 44 patients, it includes most patients with mild CV requiring rituximab treatment. The characteristics of the two populations (Table 1) showed no significant differences on epidemiology with a median age of 70 years old for the PP cohort and 66 years old for the CT cohort (ns), more than 2/3 of women and a large majority of type II cryoglobulinemia (PP: 89% and CT: 66%). We noticed the presence of 14% of type III cryoglobulinemia in the CT cohort while there was none in the PP cohort. The underlying etiologies were comparable with a predominance of patients with underlying hemopathy: 14 patients (51%) in the PP cohort (12 BCL and 2 PCD) and 18 patients (41%) in the CT cohort (14 BCL and 4 PCD). Among them, low-grade lymphoma (marginal zone lymphoma, mucosa-associated lymphoid tissue (MALT) lymphoma, lymphoplasmacytic lymphoma) were the most frequent (PP: 50% and CT: 50%) followed by monoclonal gammopathy of Undetermined Significance (MGUS) (PP: 35% and CT: 39%) and more rarely by high-grade lymphoma (PP: 14% and CT: 0%) or myeloma (PP: 0% and CT: 6%). Underlying AID occurred in 7 patients (26%) of the PP cohort and in 16 patients (36%) of the CT cohort. Among them, Gougerot-Sjögren syndrome was by far the most frequent associated condition (PP: 86% and CT: 69%) and more rarely systemic lupus erythematosus (PP: 14% and CT: 12%), rheumatoid arthritis (PP: 0% and CT: 25%), and others (anti-phospholipids syndrome (APS), periarteritis nodosa). Seven (26%) infectious diseases were reported in the PP cohort, all of them with chronic HCV, (4 with active HCV infection and 3 with non-active HCV infection) and over half (57%) were associated with an hemopathy. In the CT cohort, 15 (34%) patients presented an infectious disease with 93% of chronic HCV (8 with active HCV infection and 6 with non-active HCV infection). Only one had chronic B hepatitis infection associated with Sjögren syndrome. Among them, 3 patients (20%) suffered from associated hemopathy and 3 patients (20%) exhibited AID (rheumatoid arthritis, Sjögren syndrome). Indeed, intertwined causes (PP: 19% and CT: 23%) can be observed with low-grade lymphomas and/or autoimmune manifestations developing on a ground of chronic HCV infection. Idiopathic CV were less common and found at a comparable rate in both cohort (PP: 15% and CT: 11%).

**Table 1:** Characteristics of the two populations at treatment initiation in both cohort.

	PP cohort n = 27	CT cohort n = 44	*P*
**Median age (year) (min-max)**	70 (47–86)	66 (31–87)	ns
**Sex F (%)**	18 (67%)	32 (73%)	ns
**Type of cryoglobulinemia (%)**			ns
Type I	3 (11%)	9 (20%)	
Type II	24 (89%)	29 (66%)	
Type III	0 (0%)	6 (14%)	
**Etiology**			
Infection	3 (11%)	9 (20%)	ns
Hemopathy	9 (33%)	12 (27%)	ns
AID	6 (22%)	9 (20%)	ns
Essential	4 (15%)	5 (11%)	ns
Mix:	5 (18%)	9 (20%)	ns
*Hemopathy + infection*	*4 (15%)*	*3 (7%)*	
*Hemopathy + AID*	*1 (4%)*	*3 (7%)*	
*Infection + AID*	*0 (0%)*	*3 (7%)*	
**Median number of organ involvements (min-max)**	**4 (2–7)**	**2 (1–6)**	ns
General symptoms	19 (70%)	7 (16%)	<0.0001
Renal disease	17 (63%)	11 (25%)	0.04
Neuropathy	16 (59%)	15 (34%)	ns
Skin manifestation	24 (89%)	30 (68%)	0.04
Skin necrosis	12 (44%)	7 (16%)	0.003
Arthritis involvement	12 (44%)	21 (48%)	ns
Cardiac disease	3 (11%)	3 (7,0%)	ns
Gastro-intestinal involvement	5 (19%)	0 (0%)	ns
CNS involvement	1 (4%)	1 (2,3%)	ns
Other	4 (15%)	7 (16%)	ns
**Concomitant treatment**			
None	0 (0%)	10 (23%)	0,007
Steroids	25 (96%)	28 (65%)	0,006
Anti-HCV therapy association	1 (4%)	6 (14%)	ns
*Interferon*	*1*	*3*	
*Ribavirin*	*1*	*4*	
*DAAs*	*0*	*7*	
Methotrexate	0 (0%)	3 (7%)	ns
Hydroxychloroquinne	0 (0%)	4 (9%)	ns
Chemotherapy	3 (11%)	3 (7%)	ns
Immunosuppressive treatments	0 (0%)	1 (2%)	ns
**ICU**	4 (15%)	2 (5%)	ns
**Dialysis**	0 (0%)	1 (2%)	ns

F = female gender; AID: auto-immune disease; CNS: central nervous system; HCV: hepatitis C virus; ICU: intensive care unit; ns: not significant.

### 3.2 Prior to rituximab, PP cohort presented significantly more severe CV

As expected, both cohorts were dissimilar on CV characteristics prior to rituximab (Table 1), with more severe diseases in PP cohort. Patients in this cohort presented a median number of organs involved of 4 *vs.* 2 in the CT cohort. Furthermore, we reported a higher rate of organ involvements reflecting a more severe disease: renal involvement (63% *vs.* 25% for CT, *P* = 0.004), general symptoms (70% *vs.* 16% for CT, *P* < 0.0001) and skin necrosis (44% *vs.* 16% for CT, *P* = 0.003). Although quite rare, there were no significant differences in the prevalence of cardiac, gastro-intestinal and central nervous system (CNS) involvements, known to be life-threatening, in both groups. ICU admission and renal replacement therapy at rituximab initiation were scarce and not different in numbers in both cohorts. Biological activity was significant in the PP cohort, with a median C4 level at 0.03 mg/l and median rheumatoid factor activity at 259 UI/ml at treatment initiation. Most of the PP cohort had monoclonal (n = 13; 48%) gammopathy rather than oligoclonal (n = 6; 22%) nor absence of gammopathy. These gammopathy were more IgM (n = 9; 33%) than IgG (n = 4; 15%).

### 3.3 In this indication, plasmapheresis was feasible and safe

The median number of plasmaphereses received in the PP cohort was 8 (ranging from 1 to 33, IQR = 8). Plasmapheresis technical details are presented in Table 2. Among the 249 plasmapheresis sessions performed for the 27 patients of the PP group, adverse events were reported in only 5 of them, with two grade 2 acute pulmonary edema due to cardiac overload (quickly resolved after therapeutic adjustments) and three grade 1 vagal reaction at the end of plasmapheresis. No bleeding at puncture site, venous thrombosis nor catheter infection were reported. A central venous access was needed for 6 patients whereas peripheral access was enough for 21 patients and data were missing for the other 6 patients. The mean of extracted plasmatic volume was 2718 ml (from 1015 ml to 3558 ml) corresponding to the average of 1.05 plasma volume extracted (from 0.79 to 1.48; SD = 0.2) per session (data is missing for 5 patients). All patients except one were treated with steroids associated to the plasmapheresis and rituximab treatment. Other concomitant therapies listed in our two cohorts (Table 1) were principally anti-VHC associations (including interferon therapy, ribavirin and/or direct-acting antivirals (DAAs)) and chemotherapy according to the underlying disease. Concerning rituximab regiment, four weekly rituximab at 375 mg per m^2^ were administrated to all, except two patients at 1 g at day 1 and day 15; and one patient with no information available.

**Table 2:** Plasmapheresis technical details in the PP group (N = 27).

Patients	Number of PP sessions	TBV (L)	Ht (%)	TPV (L)	EPV (L)	SD VPE (L)	TPM	CVL	FFP	IAE
1	10	5.158	38.0	3.198	3.206	0.201	**1.003**	No	**Yes**	No
2	8	6.222	36.0	3.982	3.557	0.442	**0.893**	na	**Yes**	No
3	3	5.377	38.0	3.334	2.892	0.36	**0.868**	No	No	No
4	3	5.152	41.0	3.040	2.840	0.150	**0.934**	No	No	No
5	9	6.272	37.2	3.938	3.288	0.279	**0.835**	No	No	No
6	5	3.962	28.4	2.837	2.808	0.236	**0.990**	No	No	No
7	4	3.437	31.0	2.371	2.548	0.191	**1.074**	**Yes**	No	No
8	11	3.628	35.0	2.358	3.143	0.452	**1.333**	No	No	No
9	4	3.738	29.0	2.654	3.544	0.419	**1.335**	**Yes**	**Yes**	No
10	1	3.437	32.0	2.337	3.470	na	**1.485**	No	No	No
11	20	3.627	38.3	2.236	2.515	0.283	**1.125**	No	No	No
12	2	5.758	37.8	3.582	3.083	na	**0.861**	No	No	**Yes** (VAR)
13	10	5.802	Na	na	2.387	0.207	**NA**	na	No	No
14	10	3.639	33.0	2.438	2.637	0.323	**1.082**	No	**Yes**	No
15	4	4.784	36.6	3.033	2.393	0.804	**0.789**	**Yes**	No	No
16	18	4.038	Na	na	1.832	0.47	**NA**	na	No	No
17	2	4.119	33.3	2.750	2.953	na	**1.074**	No	No	No
18	1	3.110	31.0	2.146	2.410	na	**1.123**	No	No	No
19	10	4.386	28.3	3.144	2.519	0.317	**0.801**	**Yes**	**Yes**	No
20	7	4.145	36.7	2.624	3.220	0.376	**1.227**	No	No	No
21	10	3.171	29.3	2.243	3.190	0.264	**1.422**	No	No	**Yes** (APE + VAR)
22	2	3.540	Na	na	1.014	0.955	**na**	na	No	No
23	33	3.006	30.7	2.083	2.070	0.291	**0.994**	na	**Yes**	**Yes** (APE)
24	10	3.263	24.9	2.449	2.525	0.226	**1.031**	**Yes**	No	No
25	18	4.757	Na	na	2.156	0.415	**na**	na	No	No
26	8	4.079	29.0	2.898	na	0.958	**na**	**Yes**	No	No
27	26	3.838	30.7	2.661	2.466	0.330	**0.927**	No	No	**Yes** (VAR)
**TOTAL**	**249.00**	**/**	**/**	**/**	**/**	**/**	**/**	**6**	**6**	**4**
**MEDIAN**	**/**	**4.276**	**33.3**	**2.797**	**2.718**	**0.357**	**1.055**	**/**	**/**	**/**

- Ht is measured by blood samples analysis. TBV and TPV are calculated according to Ht, weight and height of the patient at each sessions. EPV is the volume extracted and treated by the machine at the end of the sessions then expressed in TPM (maximal objective at 1,4). All the previous parameters are the average of all the sessions per patients.

- CVL refers to the need of CVL for at least one session.

- FFP refers to the need of partial or complete replacement of albumin 4% by FFP to avoid hemorrhagic risk due to dilution coagulopathy.

- IAE refers to complications of the plasmapheresis sessions.

TBV: total blood volume (liters); Ht: hematocrit (%); TPV: total plasma volume (liters); EPV: extracted plasma volume (liters); SD: standard deviation; TPM: treated plasmatic mass; CVL: central venous line; FFP: fresh frozen plasma; IAE: iatrogenic adverse events; VAR: vagal adverse reaction; APE: acute pulmonary edema; na: not available.

### 3.4 Plasmapheresis seems to prevent flare effect and to increase time to next treatment

**Fig. 1:**
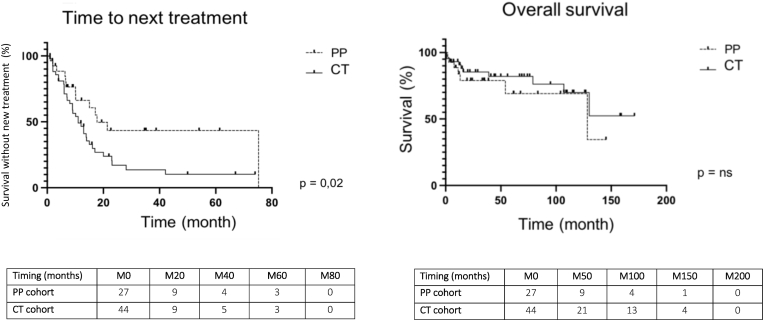
Time to next treatment (TTNT) and overall survival (OS) in both cohorts.

Even though subjects of the PP cohort had significantly more severe CV, no flare effect was observed in this group. In the other hand, 5 flares occurred in the CT cohort (Table 3). Bio-clinical presentation and outcomes are presented in Table 4. Four of them were provided with emergency plasmapheresis and two patients required a transfer to the ICU. Despite this, three of the 5 patients died, due to CV flare manifestations. The 5 patients who developed rituximab related flare-up effect had an underlying hemopathy: 2 lymphoplasmacytic lymphoma, 1 marginal zone lymphoma and 2 MGUS (one IgM and one IgG). Only one patient had an associated AID (Sjögren syndrome). There were two type II and three type I cryoglobulinemia but all subjects demonstrated monoclonal gammopathy, with a monoclonal kappa IgM in 4 out of the 5 patients*.* The rituximab regimen was 4 weekly perfusions at 375 mg per m^2^ for all patients. Three patients received the complete treatment prior CV flare manifestations, while the other two had only 1 or 2 perfusions before CV flare onset. There was no significant difference between cohorts in CV responses (Table 3). Overall response (complete responses and partial responses) occurred in 89% of the PP cohort and in 80% of the CT cohort (ns). After rituximab induction, nine patients (33%) had rituximab maintenance (systematic remote rituximab) therapy and 10 (37%) received sequential rituximab infusion (retreatment with rituximab whenever markers of disease activity occurred). With a median follow-up of 35,2 months (Fig. 1), there were no significant differences in OS with a median OS at 128,4 months in the PP cohort *vs.* not reached in the CT cohort (ns; HR = 1,52 (95% CI = 0,54–4,25)). Of note, OS at 3 years is 78,9% in PP cohort *vs.* 82,1% in CT cohort. It is noteworthy that a significantly longer TTNT was reported in the PP cohort with a median TTNT at 17.7 months *vs.* 11 months for the CT population (*P* = 0.02; HR = 0,49 (95% CI = 0,27–0,88)).

**Table 3:** Responses to treatment and outcomes in both cohorts.

	PP cohort N = 27	CT cohort N = 44	*P*
**Flare up effect**	**0 (0%)**	**5 (11%)**	**ns**
**Best response to treatment**			
Complete response	10 (37%)	21 (48%)	ns
Partial response	11 (41%)	14 (32%)	ns
Stable disease	6 (22%)	4 (9%)	ns
Persistence of biological activity after treatment	**10 (37%)**	**8 (18%)**	**ns**
Median TTNT (months)	**17.7**	**11**	**0,02**
Median OS (months)	**128,4**	**Nr**	**ns**

Best response to treatment was defined as the response achieved after rituximab therapy according to biological and clinical evaluation. OS: overall survival; TTNT: time to next treatment; ns: not significant.

**Table 4:** Flare up effect.

Patient	1	2	3	4	5	ALL/median
PP/CT cohort	**CT**	**CT**	**CT**	**CT**	**CT**	100% CT
Sex M/F	**F**	**M**	**M**	**F**	**F**	2/3
Age at Rx initiation (years)	**67**	**50**	**75**	**67**	**53**	67
Type of cryoglobulinemia	**I**	**I**	**II**	**I**	**II**	3/5 I 2/5 II
Etiology	**BCL + AID**	**BCL**	**BCL**	**MGUS**	**MGUS**	100% BCL 1/5 AI
Concomitant treatment	**CS + ChT**	**/**	**CS**	**/**	**/**	
Initial organ involvement	Pulmonary	Digital ischemia	Renal Cutaneous Neuropathy	Cutaneous necrosis	Cutaneous Arthritis	
Flare manifestations	Pulmonary with AH suspicion	Digital ischemia	Renal Cutaneous necrosis and legs ischemia	Cutaneous necrosis	Cutaneous; Arthritis	
**Biological markers**						
C3 (mg/L)	0,42	na	0,58	na	na	
C4 (mg/L)	0,02	na	0,03	na	na	
IF	IgM kappa	IgM kappa	IgM kappa	IgG lambda	IgM kappa	
RF (UI/mL)	<10	<10	125	na	na	
ICU admission	**Yes**	**No**	**Yes**	**No**	**na**	**2/5**
Flare-up management	0	**PEx**	**CS + PEx**	**PEx**	endoxan	**3/5 PEx**
TTNT	**NR**	**2 months**	**1 months**	**2 months**	**2 months**	
Death related to CV flare	**Yes**	**No**	**Yes**	**Yes**	**No**	**3/5**
OS	**1 months**	**NR**	**4 months**	**39 months**	**NR**	

Flare-up effect manifestations and outcomes for the five patients concerned in the CT cohort. BCL: B cell lymphoproliferation; AID: auto-immune disease; CS: corticosteroids; ChT: chemotherapy; na: not available; PP: plasmapheresis; OS: overall survival; TTNT: time to next treatment; NR: not reached.

## 4 Discussion

Plasmapheresis seems to be efficient and well tolerated in preventing rituximab-associated CV flare. Indeed, no CV flare was observed in the PP cohort, whereas five occurred in the CT cohort, even though patients of the PP cohort presented more severe CV features prior to rituximab treatment. Relatively to the literature, we also managed to decrease the flare effect incidence in CV patients with rituximab treatment. Interestingly, we reported a significant increase of the time to next treatment in the PP cohort. Hence, we believe our data support the use of plasmapheresis before or during rituximab treatment of moderate to severe CV.

So far, other published studies [6,11] recommended the use of plasmapheresis, for rituximab-related IgM flare prevention, only in type 1 cryoglobulinemia, if IgM levels are greater than 4 g/dl. Plasmapheresis is also recommended in a curative goal in case of type 1 cryoglobulinemia with severe renal involvement or extensive leg necrosis. In case of mixed cryoglobulinemia, curative plasmapheresis is advised if there are concomitant severe disease manifestations (membranoproliferative glomerulonephritis, leg ulcers) or highly deleterious life-threatening events such as pulmonary bleeding or intestinal vasculitis. Concerning CV flare frequency, we observe 11% of CV flare in our CT cohort. In the literature, the two recent articles focusing on the epidemiology of rituximab flare up effect in CV showed different CV flare incidence (from 3.5% [16] to 22% [17]*.*). The dissimilarities in results are mostly by the fact the first paper includes non-cryoglobulinemic vasculitis. If we compare with the number of CV (n = 50), the incidence of rituximab-related flare is 14%. It could also partially be explained by CV flare definition discrepancies, resumed in Table 5. In the present work, we have decided to define CV flare as any worsening of the initial manifestation or any new organ onset occurring during the 4 weeks after rituximab treatment initialization. Indeed, when focusing on patient No. 1 and patient No. 4, even if only one organ was damaged, mortality and morbidities have nevertheless appeared. Additionally, to definition differences, the divergences in the results may be due to the small sizes of all the populations considered.

**Table 5:** CV flare's definition according to the different papers.

	Desbois et al.	Sy-Go et al.	Present paper
Clinical deterioration	Vasculitis exacerbation in more than one organ and/or with histological proof	New organ involvement **or** autoimmune disease unexplained aggravation	New organ involvement **or** autoimmune disease unexplained aggravation
Delay after the first rituximab infusion	4 weeks	2 weeks	4 weeks

The mechanisms leading to rituximab-related flares are not fully documented. Rituximab is an anti-CD20 chimeric monoclonal antibody that is supposed to induce the killing of B-lymphocytes producing Ig and auto-antibodies, and thus avoid the release of these antibodies, via specific complement-dependent cellular depletion [18]. The mechanisms by which this treatment can lead to severe flare effects are not clearly understood. We instinctively suppose that cell lysis by rituximab infusion would lead to B-cells releasing the cryoglobulins but the mechanism seems to be more complex. The rheumatoid factor activity of the IgM seems to be a major player in this reaction [16] by its ability to interact with rituximab molecule and form new immune complexes [[19], [20], [21]]. It can explain that, without necessarily meeting the lymphoplasmacytic lymphoma criteria, all patients with CV flare displayed proof of underlying BCL with monoclonal gammopathy, almost exclusively IgM. Other processes and cellular pathways can be involved, such as IgM expression modulation on B-cells membrane surface through CD20 signaling [22, p. 20], IgG1 immunopathogenic properties [23] or IL-6 stimulated by rituximab therapy leading to rapid production of Ig [24]. Even though the tumoral burden is limited in CV, plasmapheresis, as a blood exchanger, is a secure and adapted treatment that immediately removes cryoglobulins basal level and immune complexes, reducing the consequences of their potential liberation or formation in rituximab context. There are notable differences in mechanisms leading to organ damages between type I and mixed cryoglobulinemias. Aggregation of mixed cryoglobulinemia results in immune complexes formation by activating complement and recruiting leukocytes leading to micro-inflammatory vasculitis [2]. On another side, symptoms of type I cryoglobulinemia are more induced by microcirculation obstruction due to Ig precipitation with no immune complex-mediated inflammation of blood vessels [1]. That is why clinical presentations are slightly different with more ulcers, gangrenes and skin necrosis [1]. The flare presentations also differ with slower worsening of single organ involvement (like ulcers or digital ischemia) in type I CV, which may illustrate the complexity of differential diagnosis of rituximab-related flare: natural history of CV, treatment failure and/or rituximab loss of function. Whatever, an early CV relapse may be directly related to rituximab therapy, which does not necessarily signify treatment failure and should not lead to a systematic change of treatment regimen. In our experience, plasmapheresis seems to be the best option to allow patients to safely wait until rituximab efficacy and often avoid severe complications. Nevertheless, plasmapheresis accessibility can be an issue, but CV are rare and complex diseases that need to be taken care in referring centers, which are often next to an apheresis unit.

In this retrospective analysis, data collected from patients' files were sometimes ancient and lacked of information. For instance, we were not able to use objective evaluation scales for clinical organ responses. Also, we did not have access to cryoglobulin quantification (cryocrit) for the large majority of patients while some authors suggest to use this parameter to discuss preventive plasmapheresis adjunction to rituximab therapy [19]. However, this last point reflects real-life conditions in several centers, as cryoglobulin quantification is technically difficult and is not always routinely employed. Furthermore, our report compiles all our center CV patient's data within the last twenty years and the follow-up of the corresponding treatment strategies evolution was thus rendered possible. It is to note that plasmapheresis utilization became more systematic because of patient 3, since in this case the CV flare was proven rapidly lethal. Groups comparison on this point can be difficult, as treatment strategy and supportive care evolved during the last two decades, and PP cohort correspond to newer patients. The use of corticosteroid became more systematic. DAAs development since 2005 profoundly changed HCV infection management and drastically improved its prognosis including infectious CV secondary to HCV. Immunomodulators and other targeted therapies development also enhance hemopathy management especially plasmatic dyscrasias. However, we can assume that plasmapheresis itself could potentiate rituximab efficacy with a better initial control of the disease, reduce rituximab deleterious side effects and associated risks by lowering cryoglobulin plasmatic levels.

In our opinion, and despite these weaknesses, our findings suggest that the use of preventive plasmapheresis in this indication may reduce rituximab induced CV flare frequency and extend the time to the next treatment. Given the potentially deleterious outcomes of CV flare, plasmapheresis is a simple and well-tolerate treatment that could be systematically proposed to patients with moderate to severe CV requiring rituximab therapy, without requiring cryoglobulin quantification monitoring. Larger and multicentric trials could be informative to assess stronger recommendations in CV treatment management and rituximab-related flare prevention.

## Declaration of competing interest

The authors declare that they have no known competing financial interests or personal relationships that could have appeared to influence the work reported in this paper.

## Data Availability

Data will be made available on request.
